# Cardiopulmonary resuscitation in pediatric patients under palliative home care – A multicenter retrospective study

**DOI:** 10.3389/fped.2022.1105609

**Published:** 2023-01-10

**Authors:** Emmanuel Schneck, Gisela Janßen, Vera Vaillant, Thomas Voelker, Oliver Dechert, Laura Trocan, Lioba Schmitz, Marius Rohde, Michael Sander, Holger Hauch

**Affiliations:** ^1^ Department for Anaesthesiology, Intensive Care Medicine and Pain Therapy, Justus Liebig University, Giessen, Hesse, Germany; ^2^ Palliative Care Team for Children, Heinrich-Heine-University, Duesseldorf, North Rhine-Westphalia, Germany; ^3^ Palliative Care Team for Children, Justus Liebig University, Giessen, Hesse, Germany; ^4^ Palliative Care Team for Children, Kleine Riesen Kassel, Kassel, Hesse, Germany; ^5^ Department for Pediatric Oncology and Hematology, Justus Liebig University, Giessen, Hesse, Germany

**Keywords:** emergency medial services, ethics, CPR, advanced pediatric life support, rare diseases, chronic illness, end-of-life, palliative home care

## Abstract

**Introduction:** Patients under palliative home care have special needs for their end-of-life support, which in general does not automatically include cardiopulmonary resuscitation. However, emergency medical services (EMS) respond to emergencies in children under palliative care that lead to cardiopulmonary resuscitation. To understand the underlying steps of decision-making, this retrospective, cross-sectional, multicenter study aimed to analyze pediatric patients under palliative home care who had been resuscitated. **Methods:** This study included patients from three spezialized pediatric palliative home care (SHPC) teams. The primary study parameters were the prevalence of cardiopulmonary resuscitation and the decision-making for carrying out pediatric advanced life support (PALS). Further analyses included the causes of cardiac arrest, the type of CPR (basic life support, advanced life support), the patient´s outcome, and involvement of the SHPC in the resuscitation. Descriptive statistical analysis was performed. **Results:** In total, 880 pediatric patients under palliative home care were included over 8.5 years, of which 17 patients were resuscitated once and two patients twice (overall, 19 events with CPR, 21.6 per 1,000 cases). In 10 of the 19 incidents (52.6%), cardiac arrest occurred suddenly without being predictable. The causes of cardiac arrest varied widely. PALS was performed in 78.9% of the cases by EMS teams. In 12 of 19 events (63.2%) resuscitation was performed on explicit wish of the parents. However, from a medical point of view, only four resuscitation attempts were reasonable. In total 7 of 17 (41.2%) patients survived cardiac arrest with a comparable quality of life. **Discussion:** Overall, resuscitation attempts were rare events in children under home palliative therapy, but if they occur, EMS are often the primary caregivers. Most resuscitation attempts occurred on explicit wish of the parents independently of the meaningfulness of the medical procedure. Despite the presence of a life-limiting disease, survival with a similar quality was achieved in one third of all resuscitated patients. This study indicates that EMS should be trained for advanced life support in children under home palliative therapy and SHPC should address the scenario of cardiac arrest also in early stages of palliative treatment. These results underline that advance care planning for these children is urgently needed.

## Introduction

The number of pediatric patients in need of home palliative care is increasing due to improved survival rates of severe diseases. In the United Kingdom, the prevalence of children with life-limiting conditions increased by 26.7 per 10,000 (95% CI: 26.5–27.0) in 2001/02 to 66.4 per 10,000 (95% CI: 66.0–66.8) in 2017/18 (1). Still, several diseases cannot be treated, leading to death during childhood and adolescence. In Germany, 32 specialized home palliative care (SHPC) teams are treating patients with life-limiting conditions (2, 3). The main aims of SHPC are the reduction of distressing symptoms and psychosocial guidance at home or in specialized ambulatory care facilities (4). Underlying diseases commonly define the problems and symptoms of this vulnerable group of children, which vary from neuropediatric, cardiac, and cancer diseases to metabolic disorders, asphyxia, and trauma conditions (5, 6).

Consequently, due to advanced therapies (e.g., respiratory support and new therapeutics), prolonged survival of these children is more common. However, the longer a patient in a vulnerable condition is treated, the more likely medical emergencies occur (5). Even though most crises can be anticipated and managed by the SHPC team, the number of emergency medical service (EMS) calls is increasing (5, 7). This happens mostly during potentially life-threatening situations (e.g., epileptic seizure, pneumonia with respiratory insufficiency) with a need for fast reactions. Even though SHPC offers individual recommendations in case of medical emergencies, cardiac arrest results in a challenging situation because the EMS teams are confronted with multiple problems outside of their routine practice. First, they must take care of children of any age in a life-threatening situation, ranging from newborns to young adults. Second, since caring for critically ill children is rare in the daily routine of EMS teams, a lack of practice is unavoidable (8). Third, EMS teams must rapidly understand complex information on chronic diseases, which are often also rare conditions. Last, EMS teams must decide if pediatric advanced life support (PALS) is indicated in the individual child. It cannot be taken for granted that all patients treated by SHPC have a “do not resuscitate (DNR)” order because the pediatric SHPC teams treat their patients over a long period and not only during their last weeks of life (9). For this reason, EMS teams can face patients who might be declared as palliative without a clear statement of resuscitation orders. It must be assumed that these factors contribute to EMS decision-making resulting in cardiopulmonary resuscitation (CPR) in children under home palliative care.

Limited scientific data is available investigating the prevalence and management of PALS in children treated by SHPC. Furthermore, it is unknown which factors contribute to the decision to perform PALS in these patients. For these reasons, this study aimed to identify cases of children under home palliative care who have been resuscitated to analyze the reasons for and outcomes of their resuscitation.

## Methods

This multicenter, cross-sectional, retrospective cohort study was approved by the local ethics committee [Justus-Liebig-University, Giessen, Germany (trial codex AZ176/22)]. The methods and results are reported according to the Strengthening the Reporting of Observational Studies in Epidemiology (STROBE) guidelines. Data were collected anonymously for all patients treated by the SHPC of the university hospitals of Giessen and Duesseldorf, as well as the pediatric SHPC service Kassel between April 1st, 2014 and September 30th, 2022.

### Data acquisition

Patients were identified by the responsible heads of the three SHPC teams. For this purpose, the local patient data management systems were first screened for any documentation of EMS responses and, in a second step, these cases were analyzed for performed ALS. Vice versa, patients who died were checked for documented CPR to minimize the risk of missing BLS attempts by the parents (without EMS responses).

The primary study parameter was the prevalence of cardiac arrest and the result of the decision-making process regarding life-sustaining therapies. Moreover, the type of CPR (basic life support [BLS], PALS [adult patients received adult advanced life support]), the patient´s outcome, and the inclusion of the SHPC team were analyzed as secondary study parameters. Patient characteristics included age, sex, underlying, additional diseases, and ethnic background.

**Figure 1:**
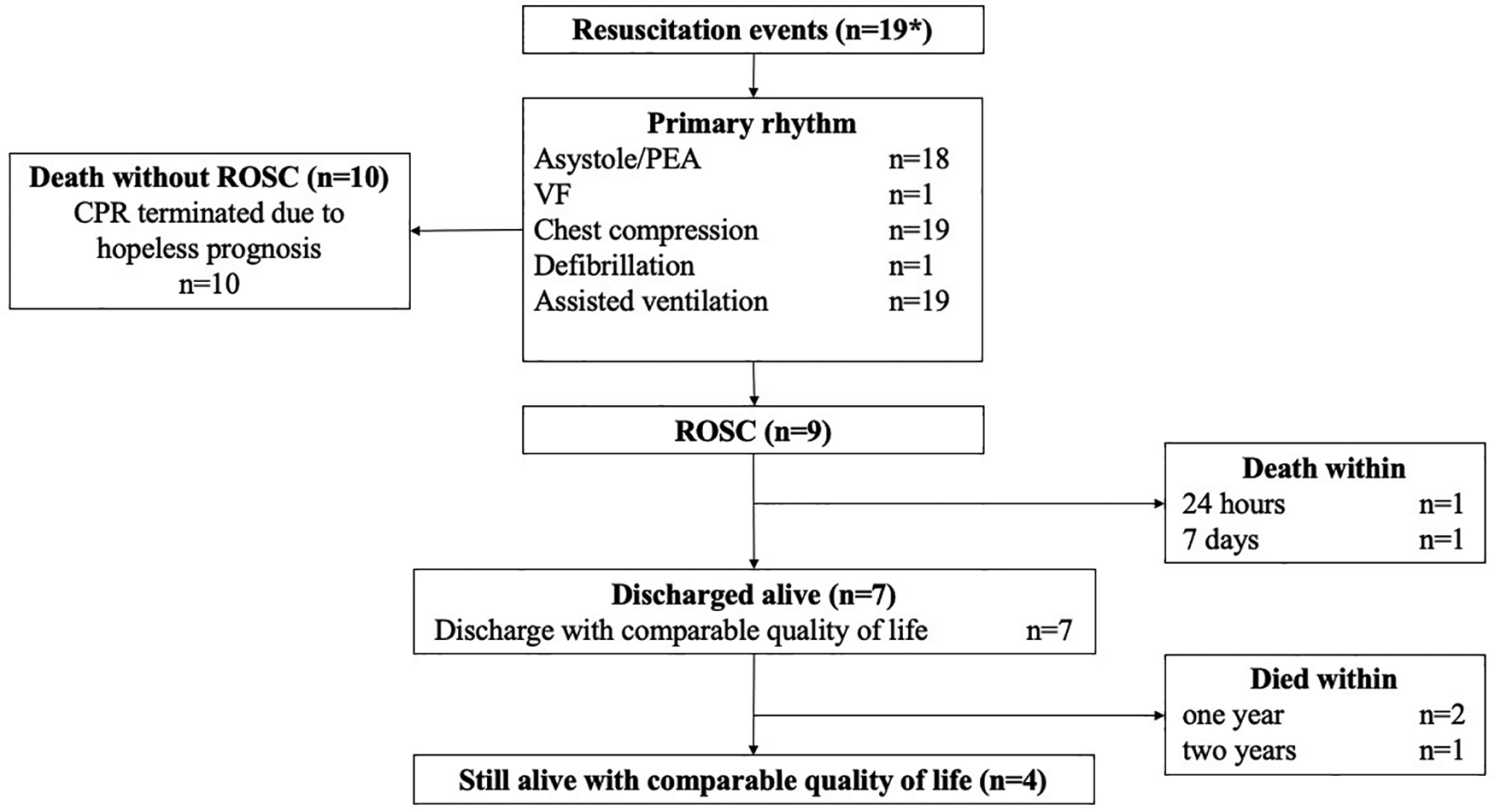
Flow chart demonstrating the resuscitation data and survival according to the utstein criteria (10). * sympolizes that 19 resuscitation events occurred in 17 patients (two patients suffered from cardiac arrest twice). Abbreviations: CPR, Cardiopulmonary resuscitation; PEA, Pulseless electrical activity; ROSC, Return of spontaneous circulation; VF, Ventricular fibrillation.

To describe the quality of life and the medical indication of the resuscitation attempt, each case was discussed with an experienced pediatrician of the SHPC team, an intensive care consultant, and a specialized SHPC nurse. Resuscitation data and survival rates are presented according to the Utstein criteria (Figure 1) (10). The P-COSCA (Pediatric Core Outcome Set for Cardiac Arrest) in children defines parameter for standardized outcome analyzes after pediatric cardiac arrest (11). Since all patients were significantly impaired in their daily life already before cardiac arrest, the post-resuscitation pediatric cerebral performance category (PCPC) alone would not have been meaningful (12, 13). Therefore, the quality of life after a successful resuscitation was assessed by the change of the PCPC. If PCPC did not worsened, the CPR was considered as successful. Resuscitation was declared as not indicated if a terminal and incurable cancer or end-stage heart or pulmonary disease were stated. Furthermore, significantly limited neurological status, a multi-professional consensus on a considerably limited quality of life, and a poor response to palliative therapy were defined as nonindicated CPR. Lastly, CPR was stated as useless if a sure sign of death or nonsurvivable severe injury was present. Otherwise, resuscitation was discussed as reasonable if a reversible cause of cardiac arrest was present or the present situation before the cardiac arrest was documented as stable with controlled symptoms (e.g., dyspnea, pain, and others).

All charts of the patients were reviewed carefully by the local teams to discuss the cases of the cardiac arrest. Therefore, any conspicuosnesses in the days before the emergency and the history of the patient were reviewed by a specialized pediatrician. The corresponding and senior author revewed these results.

### Statistical analysis

A descriptive statistical analysis was performed. Data are reported as absolute or relative values. Normally distributed data are expressed as mean ± standard deviation (SD), while non-normally distributed data are expressed as median [interquartile range (IQR)]. All statistical analyses were performed using Microsoft Excel (v.16.61.1; Seattle, WA, USA).

## Results

Overall, 880 pediatric patients under palliative home care were included over 8.5 years, of which 17 patients were resuscitated once and two patients twice (overall, 19 events with CPR, 21.6 per 1,000 cases). In these two patients cardiac arrest occurred with a time interval of five, respectively one month. The patientś characteristics are shown in Table 1.

**Table 1:** General characteristics of the study population (*N* = 17).

Age at incident (months)	98 [23.5–21.1]
Age groups (%)	
Infant (1 to 12 months)	2/17 (11.8%)
Toddler (12 months to <6 years)	5/17 (29.4%)
Schoolchild (≥6 years to <12 years)	3/17 (17.6%)
Adolescents (≥12 years to <18 years)	3/17 (17.6%)
Adults with pedatric diseases and needs (≥18 years)	4/17 (23.6%)
Sex (male)	8/17 (47.0%)
Duration of SHPC treatment (months)	7 [2.5–14.0]
Underlying diagnosis:	
Neuropediatric	9/17 (52.9%)
Oncologic	3/17 (17.6%)
Perinatal asphyxia	2/17 (11.8%)
Metabolic disorders	2/17 (11.8%)
Sequelae of severe trauma	1/17 (5.9%)
Migrant background (%)	5/17 (29.4%)
Feeding tube (%)	12/15 (80.0%, data of two patients are missing)
Implanted percutaneous central line (%)	5/15 (33.3%, data of two patients are missing)
Tracheostoma (%)	4/15 (26.7%, data of two patients are missing)

**Note.** Data are shown as absolute numbers and percentages or as a range in the case of continuous data. SHPC, Specialized home palliative care.

### Cardiopulmonary resuscitation

**Figure 2:**
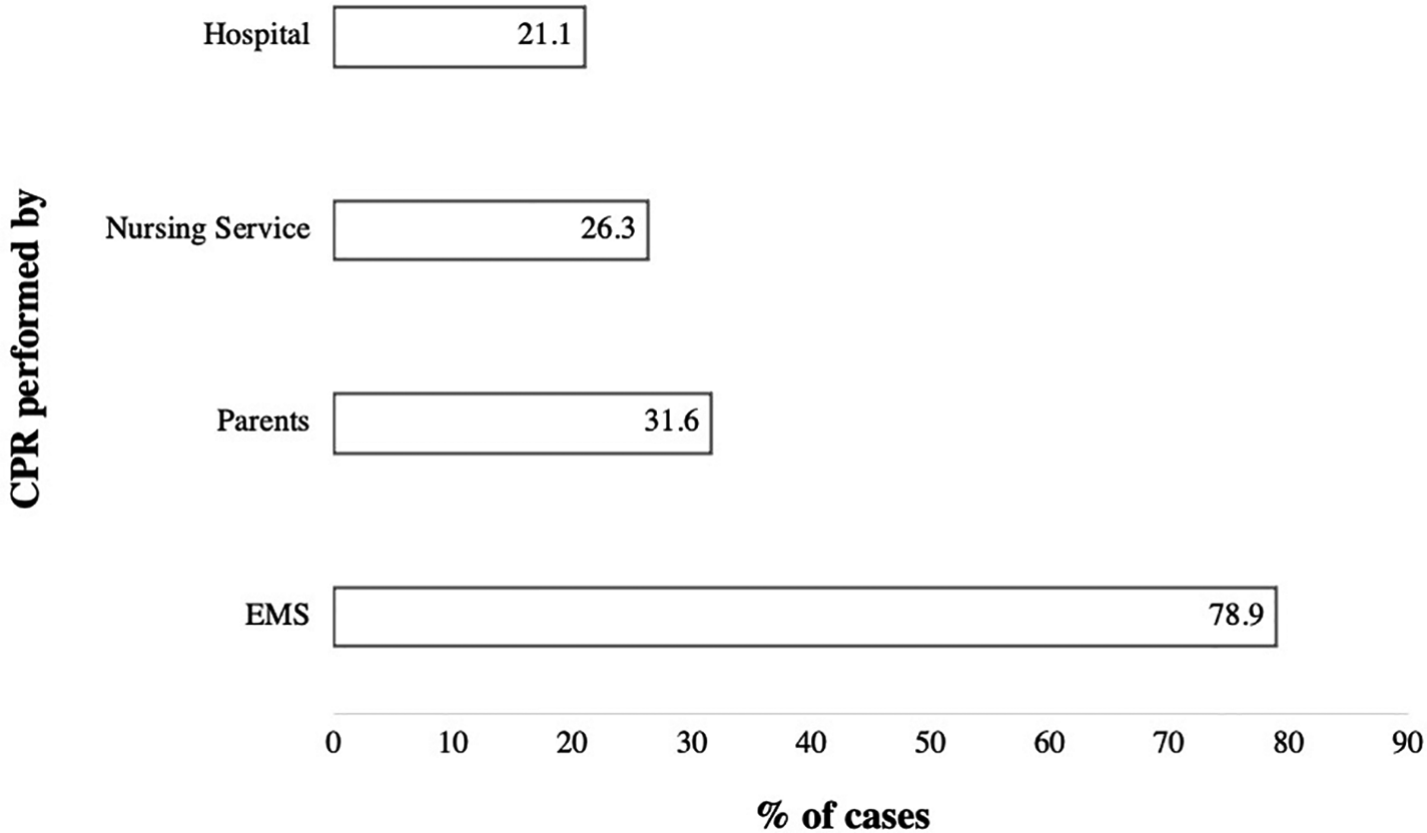
Diagram showing data from patients who were resuscitated (*N* = 19). Data are shown as percentages. Abbreviations: CPR, Cardiopulmonary resuscitation; EMS, Emergency medical service.

In the majority of events cardiac arrest occurred at home [13/19 (68.4%)], at a nursing or hospice facility (4/19 [21.1%], or at a hospital (2/19 [10.5%]. All patients who were treated in a nursing home or hospice were resuscitated by the nursing staff initially and CPR was continued by the EMS teams (Figure 2). BLS was provided by parents in 46.2% of the incidents at home, which resulted in PALS procedures by EMS teams in 84.6% of cases. In one case, the return of spontaneous circulation (ROSC) was observed after the arrival of EMS, and another patient suffered from cardiac arrest while he was being transported to the hospital by his parents. In two cases resuscitation occured during a hospital stay.

The reasons for cardiac arrest varied widely. In one case each, pulmonary embolism, pulmonary aspiration, bleeding, sepsis, and a COVID-19 infection were the suspected causes of cardiac arrest. Apnea with muscular exhaustion was observed in two cases and cerebral convulsions in three events. In two other cases, respiratory device-related problems lead to cardiac arrest, while in seven cases, a definitive cause could not be determined.

The SHPC teams were called in only six cases for advice from either the parents or the health care professionals. SHPC team members arrived during the CPR of two children and after the pronouncement of death in three further cases for crisis counseling.

Overall, in seven of 19 resuscitation attempts (36.8%) patients were discharged with comparable quality of life regarding their status prior to cardiac arrest leading to a global survival of 41.2% (*n* = 7/17; pre- and post-resuscitation PCPC 3: *n* = 2; PCPC 4: *n* = 5). The median duration of survival was 5 [2–20] months. Data presenting the survival rates are shown in Figure 1 according to Utstein style criteria (10). ROSC was achieved in 9 of 19 events (47.4%) of all performed CPR attempts.

### Decision-making

**Figure 3:**
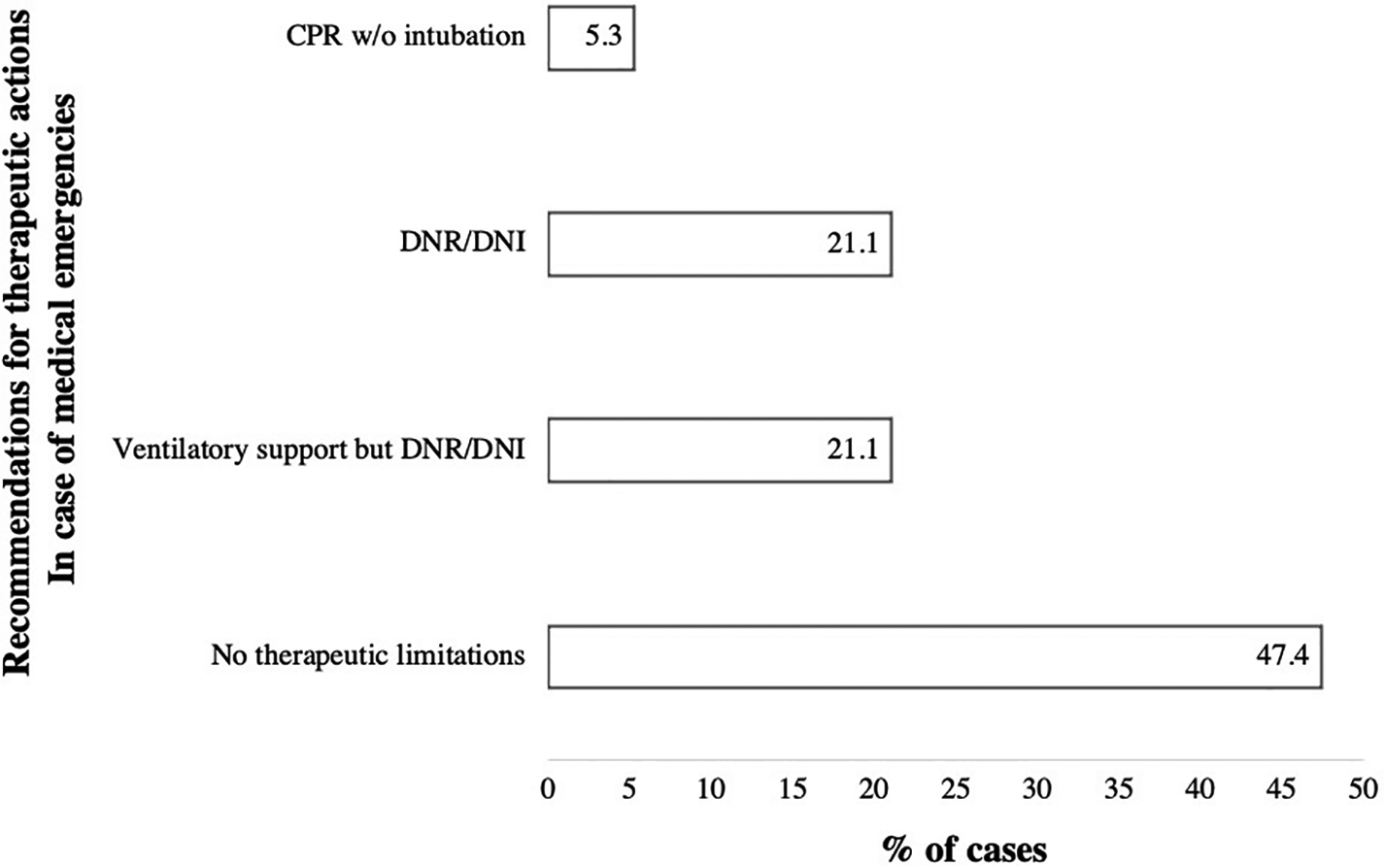
Diagram showing data on the recommendations for therapeutic action in case of medical emergencies (*N* = 19). Data are shown as percentages. Abbreviations: CPR, Cardiopulmonary resuscitation; DNI, Do not intubate; DNR, Do not resuscitate; w/o, without.

**Figure 4:**
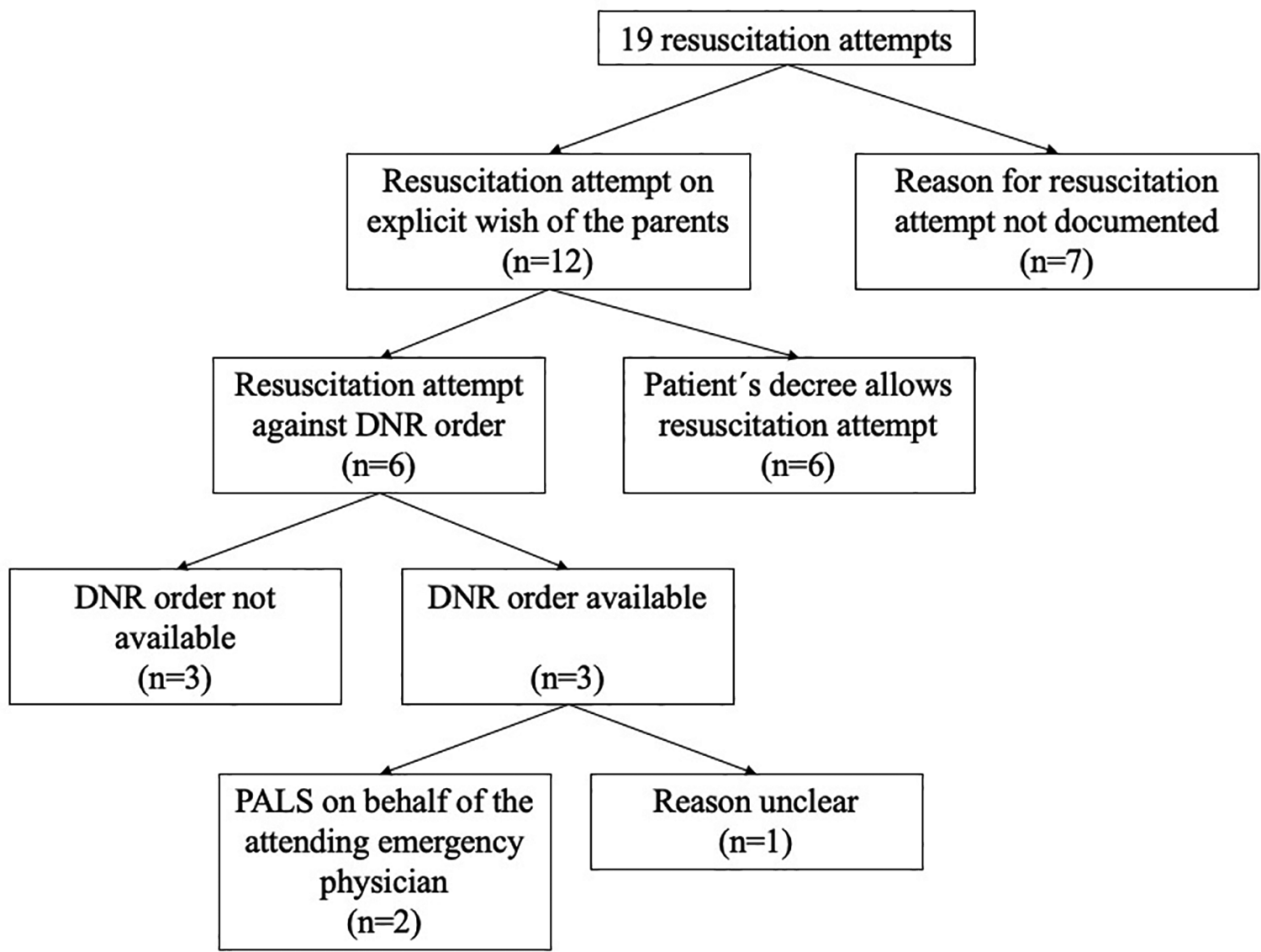
Flowchart demonstrating the decision-making for the resuscitation attempts. Abbreviations: CPR, Cardiopulmonary resuscitation; DNR, Do not resuscitate; PALS, Pediatric advanced life support.

Even though recommendations for therapeutic actions in case of emergencies were provided by the SHPC to all patients, five cases had an ongoing process of decision-making (Figure 3). In one case, no data regarding the decision-making were available. In 10 of 19 incidents (52.6%), cardiac arrest occurred suddenly without being predictable. On the other hand, in nine incidents (48.4%), the medical emergency causing the resuscitation was foreseeable. The underlying reasons of decision-making are shown in Figure 4.

From our medical point of view, only four resuscitation attempts were reasonable. Two of these patients suffered from neuromuscular disease. One patient was resuscitated due to acute lung bleeding (patient with tracheostoma), and another patient with Duchenne muscular dystrophy was diagnosed with dilatative cardiomyopathy. The patient suffered acute ventricular fibrillation while being repositioned in a nursing bed. The third patient suffered from neurometabolic disease and had a foreign body aspiration (breakfast roll). Last, a patient with a low-grade glioma with a good quality of life in a stable clinical situation had a sudden cardiac arrest after a deep vein thrombosis and suspected pulmonary embolism.

We have categorized the remaining 15 incidents (of the other 13 patients) as “not indicated CPR attempt.” Thus, we analyzed in a second step all those attempts and evaluated the outcome and if a ROSC was detected. Seven of the nonindicated CPRs showed ROSC (Table 2). All patients were evaluated, and the nonindication of CPR was established because of an unstable clinical situation, a worsening of the clinical situation, the limited success of palliative treatment, and poor quality of life (e.g., significant neurological impairment, ventilation therapy, painful symptoms). For all seven patients, it was assumed that resuscitation could not improve the patient´s situation. Six of seven patients died between a few days and 5 months after ROSC. Only one patient showed reconvalscence to the clinical condition before the cardiac arrest happened. But this one case was exceptional because the parents performed CPR (BLS) until the EMS arrived. At arrival, the EMS assessed existing vital signs. It is speculative if the patient suffered from a complete cardiac arrest.

**Table 2:** Analysis of performed medical nonindicated CPR with ROSC.

Diagnosis/Age/ Tfsl-group	Cause of medical nonindication	DNR or POLST	Suspected reason for cardiac arrest/Performed CPR/	Patient follow-up	Suspected reason for CPR-attempt
SWAN/5 years/4	Unstable situation, no improvement, little progress of palliative treatment, poor QoL	No DNR, full CPR explicit wish of parents	Seizure/BLS	Died 5 months later after a seizure	Parent´s wish/unexpected incident
Hypoxic ischemic encephalopathy/ 1 year/4	Unstable situation, no improvement, little progress of palliative treatment, poor QoL	DNR: ventilation for 10 min, no thoracic compression	Malposition of tracheal cannula/BLS/ALS	Died 1 month later after a seizure	Parent´s wish/unexpected incident/accident
SWAN/15 years/4	Unstable situation, no improvement, little progress of palliative treatment, poor QoL	No DNR: full BLS, no ALS	Seizure/BLS/ALS	Died 5 months later after a seizure	Parent´s wish
Hypoxic ischemic encephalopathy/11 months/4	Unstable situation, no improvement, little progress of palliative treatment, poor QoL	No DNR: full BLS, no ALS	Seizure/BLS (performed by parents)	Still alive	Parent´s wish/unexpected incident
Neurometabolic disorder/6 months/3	Unstable situation, no improvement, little progress of palliative treatment, poor QoL	DNR: ventilation for 15 min, no thoracic compression	Accidental disconnection of ventilation/BLS/ALS	Died a few days later in ICU	Unexpected incident/accident
Combined brain and spine injury after car accident/15 years/4	No improvement, poor QoL	No DNR, full BLS, full ALS	Sepsis/BLS/ALS	Died a few days later in ICU	Unexpected incident
SWAN/22 years/4	No improvement, poor QoL	No DNR, full BLS, full ALS	Aspiration and cardiac arrest under bronchoscopy	Died a few days later in ICU	Unexpected incident

**Note.** CPR, cardiopulmonary resuscitation, ROSC, Return of spontaneous circulation; POLST, Physician order for life-sustaining treatment; QoL, Quality of life; SWAN, Syndrome without a name; Tfsl, Together for Short Lives.

## Discussion

This retrospective data analyzes the meaning of CPR procedures in pediatric patients under palliative home care. To our knowledge, this is the first study to describe the prevalence of these cases in a multicenter pediatric cohort with life-limiting conditions. Due to the palliative treatment intention, it was expected that resuscitation events should be absolutely rare. However, CPR attempts occurred regularly.

Considering that only 0.8% of all pediatric patients without life-limiting conditions suffer from cardiac arrest in Germany, children with chronic diseases (including also palliatively treated patients) might play a relevant role (14). Various epidemiologic studies investigated the causes of pre-hospital cardiac arrest in children and showed age-dependent risks exist: While infants die from sudden infant death syndrome, toddlers and small children are at risk for severe respiratory failure and upper airway obstruction. With increasing age, the risk for major trauma events raises (15–17). The patients in the presented cohort offered various causes for cardiac arrest, which are explainable given the extensive age range and the heterogeneity of the underlying diseases. Neuropediatric disease was displayed in 52% of patients, which might explain the higher incidence of convulsions and associated apnea in our cohort. However, one meaningful limitation is that the causes of cardiac arrest cannot often be determined precisely due to the retrospective character of the study. An autopsy was performed in none of the patients.

One assumption is that patients with life-limiting conditions will not survive a cardiac arrest because of the hopeless situation, ethical issues, or the patient´s decree. This hypothesis cannot be confirmed with our data. ROSC was achieved in 47.4% of the performed CPR attempts. In summary, seven of 17 patients (41.2%) survived cardiac arrest with a comparable quality of life to the status before resuscitation. Due to the underlying severe neurological impairment of most patients in our cohort, outcome cannot be compared to pediatric cohorts. A nationwide study in Japan showed survival with good neurological quality ranged from 2.3% to 10.8% dependent on the location of cardiac arrest and survival was higher in patients who were resuscitated in a public space (15). This was mainly explainable by a strong correlation between a faster beginning of professional resuscitation in public locations. Another recent study from the Netherlands revealed an overall survival of 39% after hospital discharge and survival with favorable outcomes in 19.7% of cases, which is comparable with our cohort (18). In pediatric trauma patients, survival is generally higher; however, expectably no trauma patient was included in our study (16). Interestingly, the number of parents who performed CPR prior to EMS arrival was lower in our study (46.2% vs. 59.8% in Japan and 68% in the Netherlands) even though parents of chronically ill children are often trained and/or experienced in emergency situations. An explanation for this observation cannot be drawn from this study, but it might be a symptom of an overburdening in this critical situation. This might also explain why in 63.2% of cases patients were resuscitated against a former DNR order. Furthermore, two observations can be made in all these cases: every incident was unexpected and/or accidental and parents could not accept the acute death of their child.

It must be highlighted that EMS were the primary caregivers in pediatric patients under palliative home care, independently if the patient was at home or in a nursing facility, or a hospice. This study demonstrates the challenges for EMS teams facing cardiac arrest in children with life-limiting conditions. First, the age span is great, reaching from newborns to adults, indicating a large variability in the necessary materials and drug medications. For instance, a tracheostoma in a pediatric size is usually not available even in physician-staffed EMS. Second, the underlying diseases and causes for cardiac arrest vary to a high extent, surrogating that EMS teams must interpret a great amount of information to treat the patients adequately. Due to the low prevalence of the underlying diseases in this study, medical information could not be assessed easily. For this reason, SHPC should provide specific and individual recommendations for their patients, including the resuscitation decision of the parents. This study showed that the availability and clarity of the recommendations displayed a major problem for EMS teams. In some cases, they were not accessible, but in most cases, the parents withdrew the original decision of DNR and asked for resuscitation. In the opinion of the authors, it is almost impossible to decline the parent´s request for CPR because of the time-critical emergency setting. DNR orders and advance healthcare directives will hopefully be reliable when they are decided on as part of an advance care planning process. Furthermore, even though the patient is palliative, it cannot be assumed that the patient has a DNR order. Almost half of the patients (47.4%) had no DNR order at the time of cardiac arrest, whereas a clear DNR statement was found only in 21.1%. The other decree includes special recommendations, such as do-not-intubate orders (DNI) or minimalization of life support (e.g., ventilatory support). This is explainable by the long-term process of decision-making. Since most patients are connected early to the SHPC teams and cardiac arrest occurred already after 7 months, the parents often did not have enough time to declare a precise DNR order. This must be perceived and respected by the EMS and SHPC teams. Most importantly, SHPC should be contacted, which happened only in a minority of cases in this study. Thus, there is scope for improvement in communication between the families, SHPC, and EMS.

From an ethical point of view, no patient should receive a treatment that is not indicated and/or against their pronounced will. Nevertheless, this study showed that most resuscitation attempts were not medically indicated. This is a dilemma, and the underlying reasons and problems must be identified. Analyzing these cases with nonindicated CPR, possible improvement was only seen in one case. In all other cases, the patients died shortly after the attempt. One conclusion is that in the decision-making process, parents should think also about unexpected complications of the underlying disease (e.g., sepsis or accidents).

Strategies for the reduction of not indicated CPR events in pediatric patients under palliative home care should be discussed. First, approximately half of the events were predictable, while others occurred suddenly. This indicates that the risk of cardiac arrest should be addressed to the families already in the first weeks of SHPC care and independent of whether the situation is stable or not. In our data, CPR was also attempted if the patients were considered stable. This might help to start advanced care planning and might offer the opportunity to accelerate decision-making. Although competent and respectful communication is provided to families with a child diagnosed with a life-limiting disease, a recent Iranian study on specialized pediatricians showed that communicating the truth about the patient's status remains a major problem (19). This can be caused by physician−parents, physician−patient, or physician−physician conflicts but also by language barriers and other cultural beliefs. These issues might also have played a role in our study because 26.3% of the included families had a migrant background.

Second, the availability of medical records and recommendations must be available in emergency scenarios to support EMS. In particular, EMS should contact the SHPC team to receive focused information on the patient and allow crisis intervention. To the experience of the authors, it is helpful for the parents to be accompanied by a familiar physician of the SHPC, either in person at the scene or by phone. Our own yet unpublished data show that EMS providers felt uncomfortable with children under home palliative care and were better prepared for those emergencies after an educational intervention.

**Figure 5:**
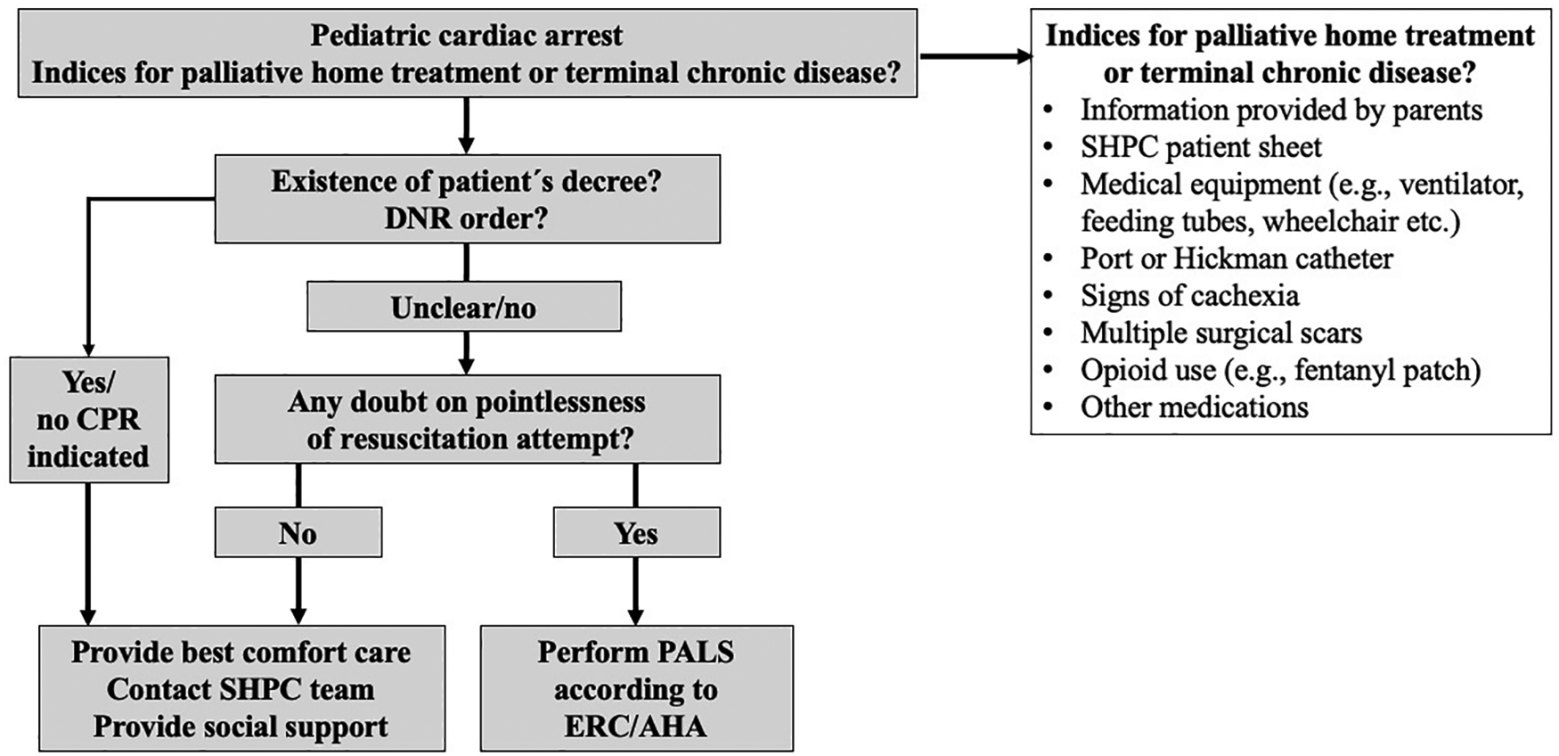
Algorithm for the management of cardiac arrest in pediatric patients under home palliative therapy. Abbreviations: AHA, American heart association; DNR, Do not resuscitate; ERC, European Resuscitation Council; PALS, Pediatric advanced life support; SHPC, Specialized palliative home care.

Third, it should be recognized that resuscitation was successful in 36.8% of cases, indicating that PALS might be indicated under special circumstances even if the child is under palliative home care. If validated indicators for survival are present, such as witnessed cardiac arrest, bystander CPR, and short duration until EMS arrival, resuscitation attempts might be justifiable (20–23). However, they must always occur with the permission of the parents (or the patient´s will if applicable). The authors recommend an individualized approach with a quick reevaluation of the situation to minimize the risk of unnecessary suffering (Figure 5). On the other hand, it must also be highlightened that 73.2% of the resuscitation attempts were unsuccessful, which can be used for the parent´s advisory and be helpful in the process of decision-making.

This study has some limitations. First, due to its retrospective structure and the long observation period, it was neither possible to interview the attending physicians or the parents concerning their views on cardiac arrest and resuscitation. Due to the severe degree of disability of the included patients, not all necessary data for the S-COSCA criteria were eligible. However, it was possible to interpret the documented patient files, which allowed conclusions to be drawn regarding the quality of life, decision-making and the resuscitation attempt. It cannot be ruled out that cases of short-lasting basic life support attempts by the parents were not recorded and might therefore not been included to the study. Second, the number of included cases is low. However, since these are very rare events, 19 documented cases in a representative cohort of more than 800 children under palliative home care are not that small in relative terms. Third, the definitive causes of death were not reported because none of the patients underwent autopsy. Last, the assessment of life quality was evaluated according to the physicianś and parentś opinions and not quantified with validated scores because these do not take a pre-existing disease with disabilities into account (24, 25).

## Conclusion

Overall, with a prevalence of 21.6 per 1,000 cases resuscitation attempts are rare events in children under palliative home care. The causes of cardiac arrest varied to high extent. Most resuscitation attempts occurred on explicit wish of the parents independently of the meaningfulness of the medical procedure. Despite the presence of life-limiting diseases, survival with an adequate quality of life was achieved in one third of all resuscitated patients. If these emergencies occur, EMS personnel are the primary caregivers. The rapid availability of resuscitation orders and the parentś decisions in the acute situation displayed major problems for the EMS teams. This study indicates that EMS should be trained for PALS in children under home palliative therapy and SHPC should address the scenario of cardiac arrest in the early stages of palliative treatment. This data provides a strong argument for starting advanced care planning in palliative home care early.

## Data Availability

The raw data supporting the conclusions of this article will be made available by the authors, without undue reservation.
